# Fasting increases susceptibility to acute myocardial ischaemia/reperfusion injury through a sirtuin-3 mediated increase in fatty acid oxidation

**DOI:** 10.1038/s41598-022-23847-w

**Published:** 2022-11-29

**Authors:** Andrew R. Hall, Qutuba G. Karwi, Sanjeev Kumar, Rachel Dongworth, Dunja Aksentijević, Tariq R. Altamimi, Kevin Timothy Fridianto, Kroekkiat Chinda, Sauri Hernandez-Resendiz, Mohammad U. Mahmood, Evangelos Michelakis, Chrishan J. Ramachandra, Jianhong Ching, Jose M. Vicencio, Michael J. Shattock, Jean-Paul Kovalik, Derek M. Yellon, Gary Lopaschuk, Derek J. Hausenloy

**Affiliations:** 1 The Hatter Cardiovascular Institute, University College London, London, UK; 2 Cardiovascular Research Centre, University of Alberta, 423 Heritage Medical Research Centre, Edmonton, AB T6G 2S2 Canada; 3 Board of Governors Regenerative Medicine Institute, Cedars-Sinai Medical Center, Los Angeles, CA USA; 4 William Harvey Research Institute, Centre for Biochemical Pharmacology, Barts and the London School of Medicine and Dentistry, Queen Mary University of London, Charterhouse Square, London, UK; 5 Cardiovascular & Metabolic Disorders Program, Duke-National University of Singapore Medical School, 8 College Road, Singapore, 169857 Singapore; 6 Department of Physiology, Faculty of Medical Science, Naresuan University, Phitsanulok, Thailand; 7 National Heart Research Institute Singapore, National Heart Centre, Singapore, Singapore; 8 Department of Medicine, University of Alberta, Edmonton, AB T6G 2B7 Canada; 9 School of Cardiovascular Medicine and Sciences, British Heart Foundation Centre of Research Excellence, King’s College London, The Rayne Institute, St Thomas’ Hospital, London, UK; 10 Yong Loo Lin School of Medicine, National University Singapore, Singapore, Singapore; 11 Chengdu University, Chengdu, China

**Keywords:** Cardiovascular biology, Cardiovascular diseases, Acute coronary syndromes, Myocardial infarction

## Abstract

Fasting increases susceptibility to acute myocardial ischaemia/reperfusion injury (IRI) but the mechanisms are unknown. Here, we investigate the role of the mitochondrial NAD^+^-dependent deacetylase, Sirtuin-3 (SIRT3), which has been shown to influence fatty acid oxidation and cardiac outcomes, as a potential mediator of this effect. Fasting was shown to shift metabolism from glucose towards fatty acid oxidation. This change in metabolic fuel substrate utilisation increased myocardial infarct size in wild-type (WT), but not SIRT3 heterozygous knock-out (KO) mice. Further analysis revealed SIRT3 KO mice were better adapted to starvation through an improved cardiac efficiency, thus protecting them from acute myocardial IRI. Mitochondria from SIRT3 KO mice were hyperacetylated compared to WT mice which may regulate key metabolic processes controlling glucose and fatty acid utilisation in the heart. Fasting and the associated metabolic switch to fatty acid respiration worsens outcomes in WT hearts, whilst hearts from SIRT3 KO mice are better adapted to oxidising fatty acids, thereby protecting them from acute myocardial IRI.

## Introduction

Metabolic substrate utilisation and efficiency are known to influence outcomes in relation to acute myocardial ischaemia/reperfusion injury (IRI)^1,2^, with worse outcomes^3^ associated with fatty acid respiration (as opposed to glucose oxidation) due to less efficient utilisation of oxygen per ATP molecule produced^4,5^. Fasting promotes fatty acid respiration through a reduction in circulating insulin and glucose levels, and a corresponding increase in circulating fatty acids. These findings may have clinical implications given that acute myocardial infarction (AMI) occurs more frequently in the morning, and the fact that patients are fasted overnight for surgery the next morning^6^. As such, in this study, we sought to understand the molecular mechanisms behind this fasting-induced switch in metabolism.

The NAD^+^-dependent mitochondrial deacetylase, Sirtuin-3 (SIRT3) is known to influence respiratory substrate utilisation, although differing effects on fatty acid oxidation have been reported in an organ-specific manner. For instance, SIRT3 KO mice show increased acetylation leading to inhibition of fatty acid metabolism in the liver^7^, whereas maturation of the neonatal to adult heart increases fatty acid metabolism^8–10^. SIRT3 is reported to mediate protection in the cardiovascular system against hypertension^11^ and cardiac hypertrophy^12–16^ although the outcomes associated with cardioprotection are unclear. In the setting of acute myocardial IRI, some studies have reported SIRT3 to be beneficial^17,18^, whilst others have reported no effects^19^.

Given that fasting upregulates SIRT3 activity^20^, it is unclear how starvation influences cardiac energy preference and outcomes following acute myocardial IRI. Therefore, we investigated the role of SIRT3 as a potential mediator of the fasting-induced increase in susceptibility to acute myocardial IRI.

## Methods

### Animal models

All animal experiments were conducted in accordance with the Animals (Scientific Procedures) Act 1986 published by the UK Home Office and the Guide for the Care and Use of Laboratory Animals published by the US National Institutes of Health 1996. All experiments were conducted under ethical approval of University College London and reported in accordance with the ARRIVE guidelines. All laboratory reagents were purchased from Sigma, Poole, UK, unless otherwise stated. Global Het SIRT3 KO mice were a kind gift from Dr David Gius (Vanderbilt University) and were bred to generate SIRT3 KO and WT littermates on a C57BL/6J background. Mice were housed at 22 °C with 12-h light–dark cycles and constant access to food (standard chow diet) and water. Experiments were conducted on littermate mice aged between 8 and 12 weeks. Based upon genotype, mice were randomised to be either fasted or fed. Fasted mice had food removed for 16–20 h prior to experimentation (by transfer to a new cage to ensure no residual food was available), which spanned the whole of the dark phase of the light/dark cycle.

### In vivo models

WT and SIRT3 KO fed and fasted mice underwent in vivo acute myocardial IRI comprising open-chest surgery by occlusion of left anterior descending coronary artery for 45 min followed by reperfusion for 24 h. Anaesthesia was by inhaled isoflurane (1.5–2.0% vaporised in 1.5 l/min oxygen). Analgesia was provided by buprenorphine (Vetergesic, Alstoe Animal Health, York, UK) 0.1 mg/kg intramuscular at 0, 6 and 24 h post-surgery^21^. After 24 h, mice were sacrificed by i.p. injection of pentoject with heparin, MI size and area-at-risk (AAR) were determined by dual staining with triphenyl-tetrazolium chloride (TTC) and Evans blue. Quantification was performed using ImageJ planimetry (NIH Image, Bethesda, MD, USA), and MI size was expressed as percentage of AAR (%IS/AAR).

Blood glucose measurements were made in conscious WT and SIRT3 KO mice under fed and fasted conditions. Blood was drawn from the tail vein and assessed using a commercially available glucometer (Accu-Chek, Roche). Circulating blood fatty acids were measured in plasma samples following manufacturer’s instructions (Abcam kit ab65341). Circulating fatty acid concentrations were then normalised to plasma protein levels, which were quantified using the BCA protein quantification assay.

### Ex vivo models

Acute myocardial IRI was assessed via Langendorff-perfused hearts from WT and SIRT3 KO (fed and fasted) mice. Mice were sacrificed by i.p. injection of pentoject with heparin. Hearts were perfused with standard Krebs–Henseleit buffer (in mM—NaCl 118, NaHCO_3_ 25, KCl 4.7, MgSO_4_·7H_2_O 1.22, KH_2_PO_4_ 1.21, CaCl_2_·2H_2_O 1.84) supplemented with either 11 mM glucose, or 1.2 mM palmitate and 5 mM glucose (concentrations which allow either glucose or fatty acid respiration to dominate)^22,23^. Palmitate was conjugated to 3% bovine serum albumin (BSA) as described previously^24^. Briefly, 1.2 mM palmitate was dissolved in 40% ethanol and 60% H_2_O and heated to evaporate the ethanol. 3% fatty-acid free BSA was dissolved in standard Krebs–Henseleit buffer (containing 5 mM glucose) and warmed to 37 °C. Palmitate was conjugated to the BSA via constant stirring for 1 h at 37 °C before being dialysed overnight (membrane with a 12–14 kDa cut-off) in 4 volumes of Krebs–Henseleit buffer at 4 °C. Buffers were then gassed and warmed prior to perfusion through respective hearts. Hearts were perfused for an initial 25 min, prior to 35 min global ischaemia, followed by 2 h reperfusion. Hearts were stained using TTC, and MI size (expressed as a % of whole heart) was quantified as described above.

Myocardial energy metabolism in IRI was assessed using an isolated working heart model, as described previously^9,25,26^. Briefly, hearts were quickly collected and perfused under aerobic conditions for 30 min with Krebs‐Henseleit solution containing 2.5 mM Ca^2+^, 5 mM [U‐14C] glucose and 1.2 mM [9,10‐3H] palmitate pre‐bound to 3% albumin. The hearts were then subjected to 20 min of global no-flow ischaemia followed by 40 min of aerobic reperfusion. Glucose and palmitate oxidation rates were measured by simultaneously collecting ^14^CO_2_ and ^3^H_2_O produced from the oxidation of [U‐^14^C] glucose and [9,10‐^3^H] palmitate, respectively. At the end of perfusion, the hearts were quickly frozen with tongs cooled to the temperature of liquid N_2_. Cardiac work was calculated as the product of peak systolic pressure (minus 11.5 mmHg preload pressure) and cardiac output. The cardiac ATP production rate was calculated based on the oxidation rate of palmitate and glucose, considering that the metabolism of one molecule of glucose and palmitate generates 31 and 104 molecules of ATP, respectively. Therefore, the following equations were used:

$$Cardiac\;ATP\;production\;rate\;from\;glucose = cardiac\;glucose\;oxidation \, rate\,* \, 31$$

$$Cardiac\;ATP\;production\;rate\;from\;palmitate = cardiac\;palmitate\;oxidation\;rate \, * \, 104$$

### Metabolomic analyses

Hearts were rapidly excised from fed and fasted WT and SIRT3 KO mice. Hearts were rapidly washed in ice cold PBS to remove ventricular blood, and then snap frozen in liquid nitrogen.

Tissue samples were thawed on ice, weighed and diluted to 50 mg tissue per mL of homogenate using an ice-cold 50% acetonitrile/0.3% formic acid aqueous solution. Tissues were homogenized on a bead mill at 4 °C. 100 µL of tissue lysate was extracted with 800 µL methanol. The methanol extract (100 µL) was dried using nitrogen gas, and derivatised using 1 M HCl in methanol (100 µL). The reactions were again dried and reconstituted with 80% methanol before being injected into the mass spectrometer for analysis. Acylcarnitines were quantified using Flow Injection Analysis (FIA)-tandem Mass Spectrometry (FIA-MS) on the Agilent 6430 Triple Quadrupole (TQ)-MS (Agilent Technologies, CA, USA) with 80/20 methanol/water as mobile phase at 0.4 mL/min, and injection volume of 2 µL. Data acquisition and analysis were performed on Agilent MassHunter Workstation B.06.00 Software.

Frozen, weighed, and pulverized hearts were subject to methanol/ water/ chloroform dual phase extraction adapted and described previously^27,28^. The upper aqueous phase was separated from the chloroform and protein fractions. 20–30 mg chelex-100 was added to chelate paramagnetic ions, vortexed and centrifuged at 3600 RPM for 1 min at 4 °C. The supernatant was then added to a fresh Falcon tube containing 10 µL universal pH indicator solution followed by vortexing and lyophilisation. Dual-phase-extracted metabolites were reconstituted in 600 µL deuterium oxide (containing 8 g/L NaCl, 0.2 g/L KCl, 1.15 g/L Na_2_HPO_4_, 0.2 g/L KH_2_PO_4_ and 0.0075% w/v trimethylsilyl propanoic acid (TSP)) and adjusted to pH ≈ 6.5 using 1 M hydrochloric acid and/or 1 M sodium hydroxide (< 5 µL of each) prior to vortexing. The solution was transferred to a 5 mm NMR tube (Norel Inc., USA) and then analysed using a Bruker Avance III 400 MHz (9.4 T) wide-bore spectrometer (Bruker, Germany) with a high-resolution broadband spectroscopy probe at 298 K. A NOESY 1D pulse sequence was used with 128 scans, 2 dummy scans, total repetition time 6.92 s, sweep width of 14 ppm and an acquisition duration of 15 min. Data were analysed using TopSpin software version 2.1 (Bruker, Germany), FIDs were multiplied by a line broadening factor of 0.3 Hz and Fourier-transformed, phase and automatic baseline-correction were applied. Chemical shifts were normalised by setting the TSP signal to 0 ppm. Peaks of interest were initially integrated automatically using a pre-written integration region text file and then manually adjusted where required. Assignment of metabolites to their respective peaks was carried out based on previously obtained in-house data, confirmed by chemical shift, NMR spectra of standards acquired under the same conditions and confirmed using Chenomx NMR Profiler Version 8.1 (Chenomx, Canada). Peak areas were normalized to the TSP peaks and metabolite concentrations quantified per gram tissue wet weight. Intracellular concentration of NADH, ATP + ADP, phosphocreatine, creatine, lactate, succinate, fumarate, carnitine, phosphocholine, choline, acetyl carnitine, acetate, aspartate, glutamine, glycine, alanine was analysed. The fold change with respect to the control group was then calculated for each metabolite.

### Mitochondrial studies

Cardiac mitochondria were isolated as described previously^29,30^. Mitochondrial respiration was assessed by measuring oxygen consumption in an Oxytherm (Hansatech, UK) with a magnetic stirrer at 25 °C. After quantification of mitochondrial protein concentration, all mitochondrial samples were normalized to a standard protein concentration of 5 µg/µL. Seventy µL of mitochondria (equivalent to 350 µL of mitochondrial protein) were added to 230 µL of Miro 5 buffer (in mmol/L, EGTA 0.5, MgCl_2_·6H_2_0 3, K-lactobionate 60, Taruine 20, KH_2_PO_4_ 10, HEPES 20, Sucrose 110, fatty acid free BSA 1 g/L, pH 7.0 with KOH) with 10 mmol/L glutamate, 10 mmol/L malate, 10 mmol/L Na pyruvate to stimulate Complex I respiration (State 2 respiration). State 3 respiration was stimulated by the addition of 0.5 mmol/L ADP, leak respiration through the addition of 0.25 mmol/L oligomycin, and maximal uncoupled respiration with sequential additions of 2 μmol/L FCCP. Finally, 5 μmol/L antimycin A was added to inhibit all respiration and assess non mitochondrial loss of O_2_ from the chamber. Oxygen consumption was expressed as nMol O_2_/min/mg of mitochondrial protein.

For western blot analysis, mitochondrial fractions were lysed in ice‐cold mitochondrial IP (MIP) buffer (1% n‐dodecyl‐β‐d‐maltoside, 0.5 mM EDTA, 150 mM NaCl, 50 mM Tris–HCl, (pH 7.4), 10 mM nicotinamide, 500 nM trichostatin A) containing a protease inhibitor cocktail (Roche)^31^ prior to separation by SDS PAGE on a 10% gel (Nu-Page, Invitrogen). Membranes were probed for pan-acetylation (Cell signalling #9441), VDAC (Cell signalling #4866), succinate dehydrogenase (Abcam ab154974), mitochondrial respiratory complexes (Abcam ab110413).

### Statistical analysis

Results were analysed using an unpaired t-test, One-Way Analysis of Variance, or Two-Way Analysis of Variance where appropriate, followed by a Tukey post-test. Statistical significance was achieved when P < 0.05.

## Results

**Figure 1:**
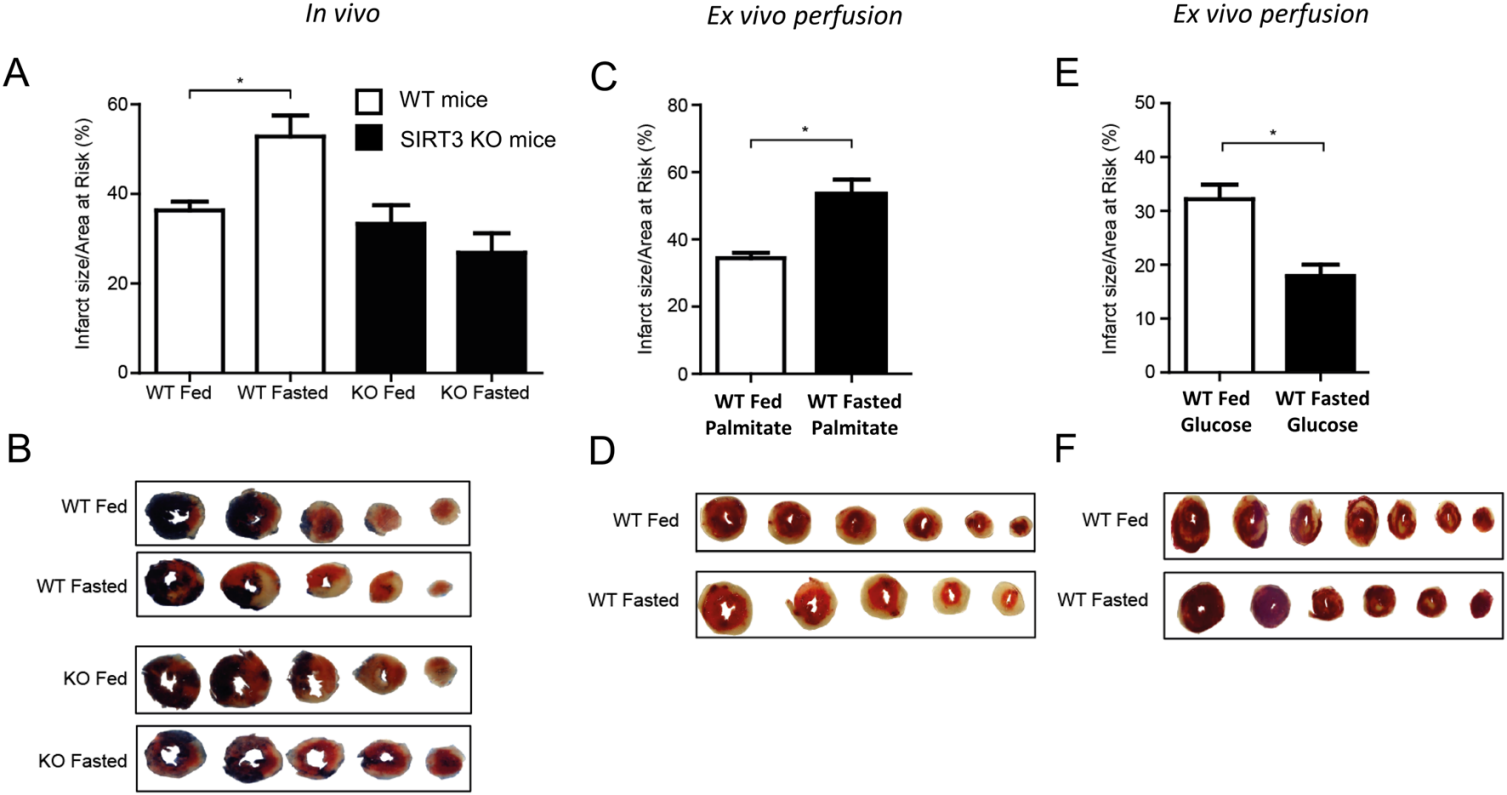
Fasting increases myocardial infarct size in WT, but not SIRT3 KO mice, with outcome dependent upon primary metabolic fuel. (**A**) Fasting increases infarct size in WT hearts, but not SIRT3 KO hearts in vivo. IS as a percentage of the area-at-risk (AAR). N = 6/group. Error bars indicate S.E.M. One-Way ANOVA *P ≤ 0.05. (**B**) Representative images of short-axis heart slices following dual staining with Evan’s blue and tetrazolium to stain the AAR and areas of infarction, respectively. (**C**) Fasting increases infarct size in ex vivo WT hearts perfused with 1.2 mM Palmitate, 5 mM Glucose. IS as a percentage of the area-at-risk (AAR). N = 6/group. Error bars indicate S.E.M. One-Way ANOVA *P ≤ 0.05. (**D**) Representative images of short-axis heart slices following staining with tetrazolium to stain the areas of infarction. (**E**) Fasting decreases infarct size in ex vivo WT hearts perfused with 11 mM Glucose. IS as a percentage of the area-at-risk (AAR). N = 6/group. Error bars indicate S.E.M. One-Way ANOVA *P ≤ 0.05. (**F**) Representative images of short-axis heart slices following staining with tetrazolium to stain the areas of infarction.

In fed animals, no differences in MI size were observed in vivo, between WT and SIRT3 KO mice (36.3% (± 2.0) vs 33.3% (± 4.1) p > 0.05, Fig. 1A,B); however, fasting significantly increased infarct size in WT mice, with no changes observed in the SIRT3 KO fasted mice (52.8% (± 4.7) vs 26.9% (± 4.3), p < 0.05, Fig. 1A,B). Concurrently, fasting significantly decreased circulating blood glucose levels, trending to increasing circulating fatty acids. Importantly, these changes in circulating metabolites were to the same extent in both WT and SIRT3 KO mice (Table 1).

**Table 1:** Fasting significantly reduces circulating glucose in starved WT and SIRT3 KO mice.

	WT fed	WT fasted	SIRT3 KO fed	SIRT3 KO fasted
Circulating glucose (mM)	9.7 (± 1.5)^†^	4.20 (± 0.5)^†^	9.6 (± 1.2)^•^	4.3 (± 0.9)^•^
Circulating free fatty acids (nMol/mg plasma protein)	0.148 (± 0.03325)	0.267 (± 0.02271)	0.193 (± 0.02402)	0.277 (± 0.07000)

(Data are mean ± SEM). ^†,•^p < 0.05 One-Way ANOVA with a Tukey post-test comparing within genotype. N = 5–7.

To understand the specific role that metabolic fuel sources have on infarct size, ex vivo WT and SIRT3 KO hearts from fed and fasted mice were perfused with either high glucose (11 mM) or palmitate (1.2 mM) plus low glucose (5 mM) (a concentration which allows fatty acid respiration to dominate to mimic the in vivo fasted conditions^32^). When WT hearts were perfused predominantly with fatty acids to mimic the in vivo changes in metabolism, fasting increased infarct size (Fig. 1C,D), however, when WT hearts were perfused solely with glucose, fasting decreased infarct size (Fig. 1E,F). No changes were observed in SIRT3 KO mice when comparing fed vs fasted state, regardless of metabolic substrate perfusion (Supplementary Fig. 1). Therefore, fasting worsens cardiac infarction in WT mice in vivo, which can be replicated ex vivo by forcing fatty acid cardiac metabolism. Interestingly, SIRT3 KO mice appear resistant to changes in outcome driven by changes in fatty acid perfusion.

**Figure 2:**
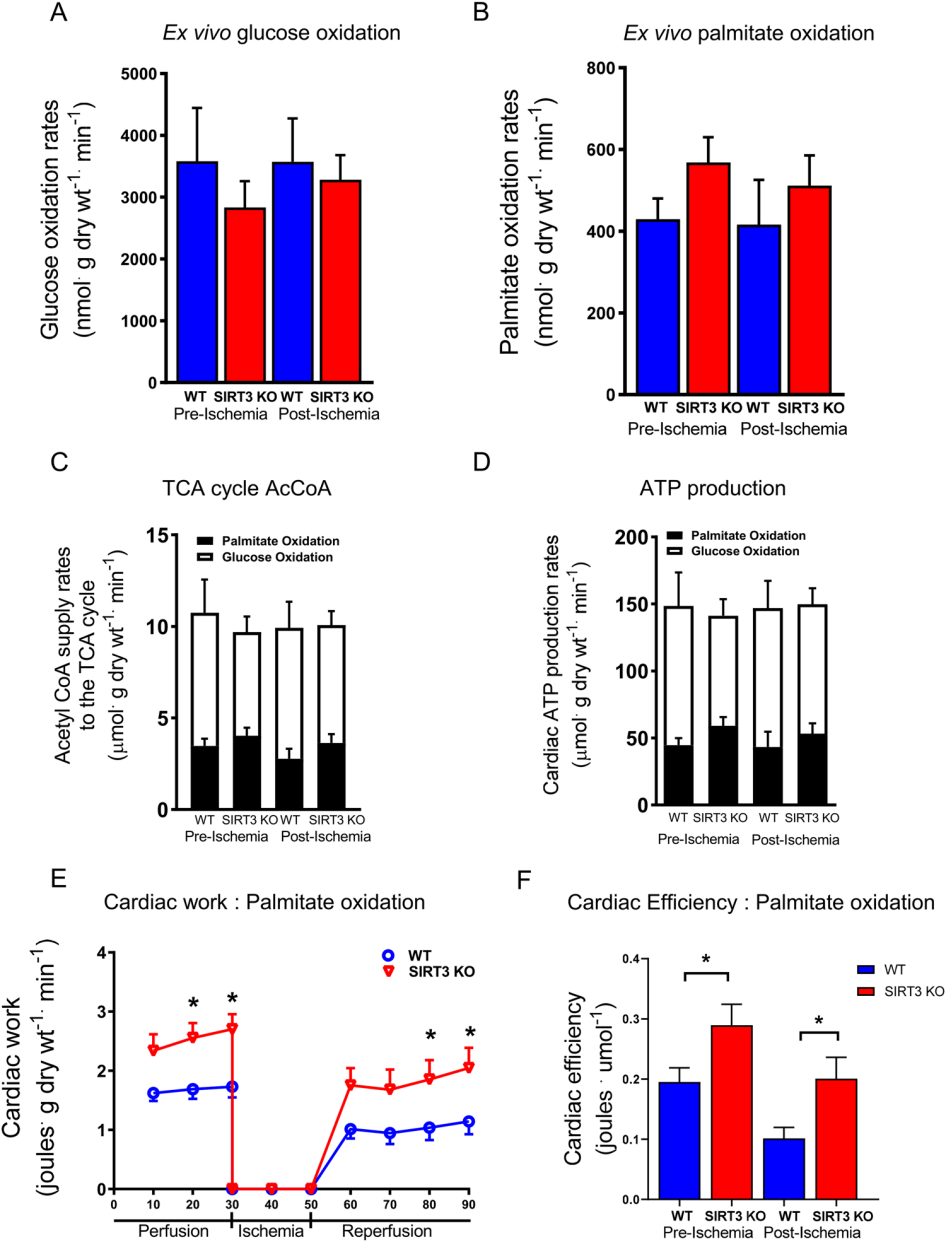
Glucose and fatty acid oxidation rates, and cardiac efficiencies in ex vivo hearts from fasted WT and fasted SIRT3 KO mice perfused with 5 mM glucose and 1.2 mM palmitate and subjected to simulated ischemia–reperfusion. (**A**) Glucose oxidation rates in hearts pre- and post- ischemia from fasted WT and SIRT3 KO mice hearts. N = 9 for WT starved, N = 7 for SIRT3 KO starved. Error bars indicate S.E.M. One-Way ANOVA, P > 0.05. (**B**) Palmitate oxidation rates in hearts pre- and post-ischemia from fasted WT and SIRT3 KO mice hearts. N = 9 for WT starved, N = 7 for SIRT3 KO starved. Error bars indicate S.E.M. One-Way ANOVA, P > 0.05. (**C**) Rates of Acetyl CoA incorporation into the TCA cycle in hearts pre- and post- ischemia from fasted WT and SIRT3 KO mice hearts. N = 9 for WT starved, N = 7 for SIRT3 KO starved. Error bars indicate S.E.M. One-Way ANOVA, P > 0.05. (**D**) Rates of ATP production in hearts pre- and post-ischemia from fasted WT and SIRT3 KO mice hearts. N = 9 for WT starved, N = 7 for SIRT3 KO starved. Error bars indicate S.E.M. One-Way ANOVA, P > 0.05. (**E**) Hearts from fasted SIRT3 KO mice produce more contractile work prior to, and post-ischemia, compared to fasted WT mice when perfused with palmitate as the primary metabolic fuel. N = 9 for WT starved, N = 7 for SIRT3 KO starved. Error bars indicate S.E.M. Two-Way ANOVA, *P ≤ 0.05. (**F**) Hearts from fasted SIRT3 KO mice are metabolically more efficient pre-, and post-ischemia than hearts from fasted WT mice. N = 9 for WT starved, N = 7 for SIRT3 KO starved. Error bars indicate S.E.M. One-Way ANOVA, *P ≤ 0.05.

Ex vivo metabolic characterisation of fasted WT and SIRT3 KO hearts perfused with fatty acids and low glucose prior to ischaemia and during reperfusion revealed no significant differences in either glucose (3581 ± 862 vs 2833 ± 427 nmol/g dry weight/min p > 0.05, Fig. 2A) or palmitate oxidation rates (429 ± 50.6 vs 568 ± 62.1 nmol/g dry weight/min, p > 0.05, Fig. 2B). Perhaps this is not surprising given the heart is an omnivore and can respire the predominant metabolic substrate available. Similar trends were observed in the generation of acetyl-CoA for break down in the TCA cycle and ATP production in the respiratory chain either prior to ischaemia, or during reperfusion (Fig. 2C,D).

Interestingly, despite similar oxidation rates, SIRT3 KO hearts had a significantly increased work capacity vs WT littermates under these conditions and a better recovery post-ischaemia compared to the WT hearts (Fig. 2E). As emphasised in Fig. 2F, SIRT3 KO mice were more efficient at converting the energy source available to contractile work done, implying that the SIRT3 KO mice are better adapted and more efficient at respiring fatty acids when fasted.

**Figure 3:**
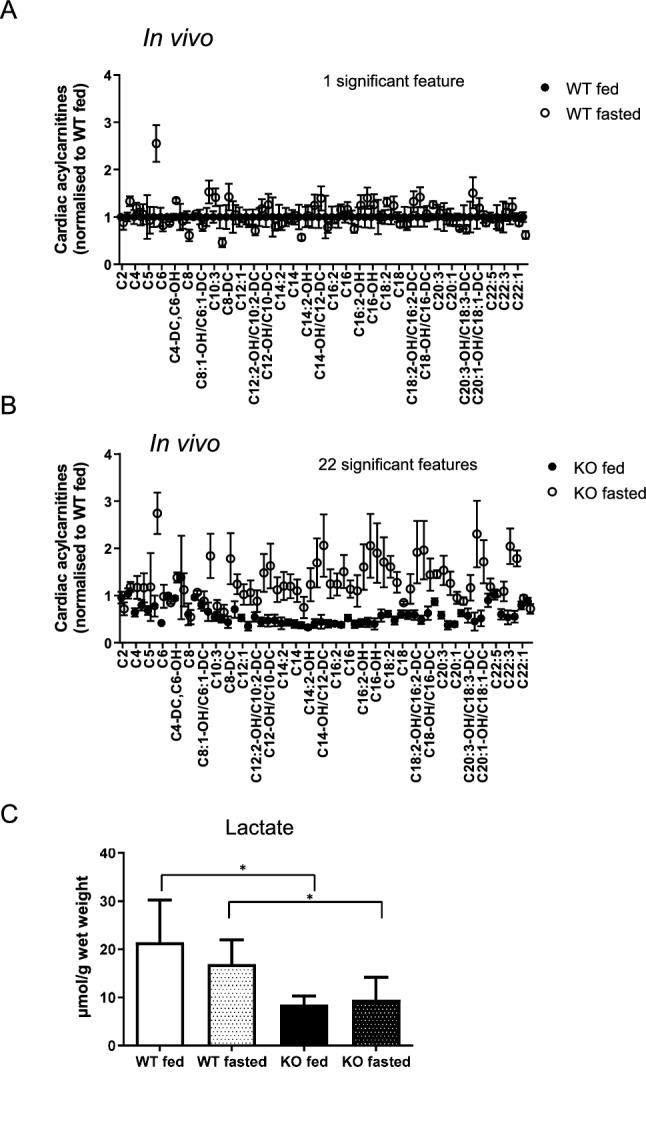
SIRT3 KO mice adapt better to oxidise fatty acids under fasted conditions, whilst WT hearts have a low degree of metabolic adaptation and preferentially respire glucose. (**A**) Profile of in vivo cardiac fatty acids from fed and fasted WT mice, with one significantly different feature (analysed by Two Way ANOVA, n = 6 for WT fed, n = 7 for WT fasted, *P ≤ 0.05). (**B**) Profile of in vivo cardiac fatty acids from fed and fasted SIRT3 KO mice, with 22 significantly different features (analysed by Two Way ANOVA, n = 6 for SIRT KO fed, n = 7 for SIRT KO fasted *P ≤ 0.05). (**C**) Profile of in vivo lactate is significantly higher in WT fed and WT fasted hearts vs hearts from fed and fasted SIRT3 KO mice (analysed by Two Way ANOVA with Tukey post-test, n = 4 for WT fed, n = 4 for WT fasted, n = 3 for SIRT3 KO fed, n = 4 for SIRT3 KO fasted *P ≤ 0.05).

To confirm ex vivo observations around metabolic substrate adaptation, the profile of cardiac fatty acids was analysed by mass spectrometry and TCA metabolites by NMR in snap frozen in vivo hearts. Analysis revealed limited variation in acylcarnitine profiles between WT fed vs WT fasted mice (Fig. 3A) with one significantly different feature. In contrast, a comparison between fed and fasted SIRT3 KO hearts, revealed 22 significant features (Fig. 3B). ^1^H NMR spectroscopy analysis of metabolites from hearts snap frozen in vivo revealed higher lactate in WT fed and fasted hearts (vs SIRT3 KO hearts) (Fig. 3C), with no other significant changes in other metabolites observed (Supplementary Table 1). Together these data suggest the SIRT3 KO heart had a greater adaptation to starvation, with a greater degree of profile change, whilst the WT hearts consistently contained more lactate than SIRT3 KO hearts. Whilst lactate amount per se does not conclusively demonstrate a higher level of glycolysis from WT hearts, it indicates a tendency towards a higher level of glycolysis. Together, these data suggest that the absence of SIRT3 allows a greater adaptation to the metabolic switches induced by starvation.

**Figure 4:**
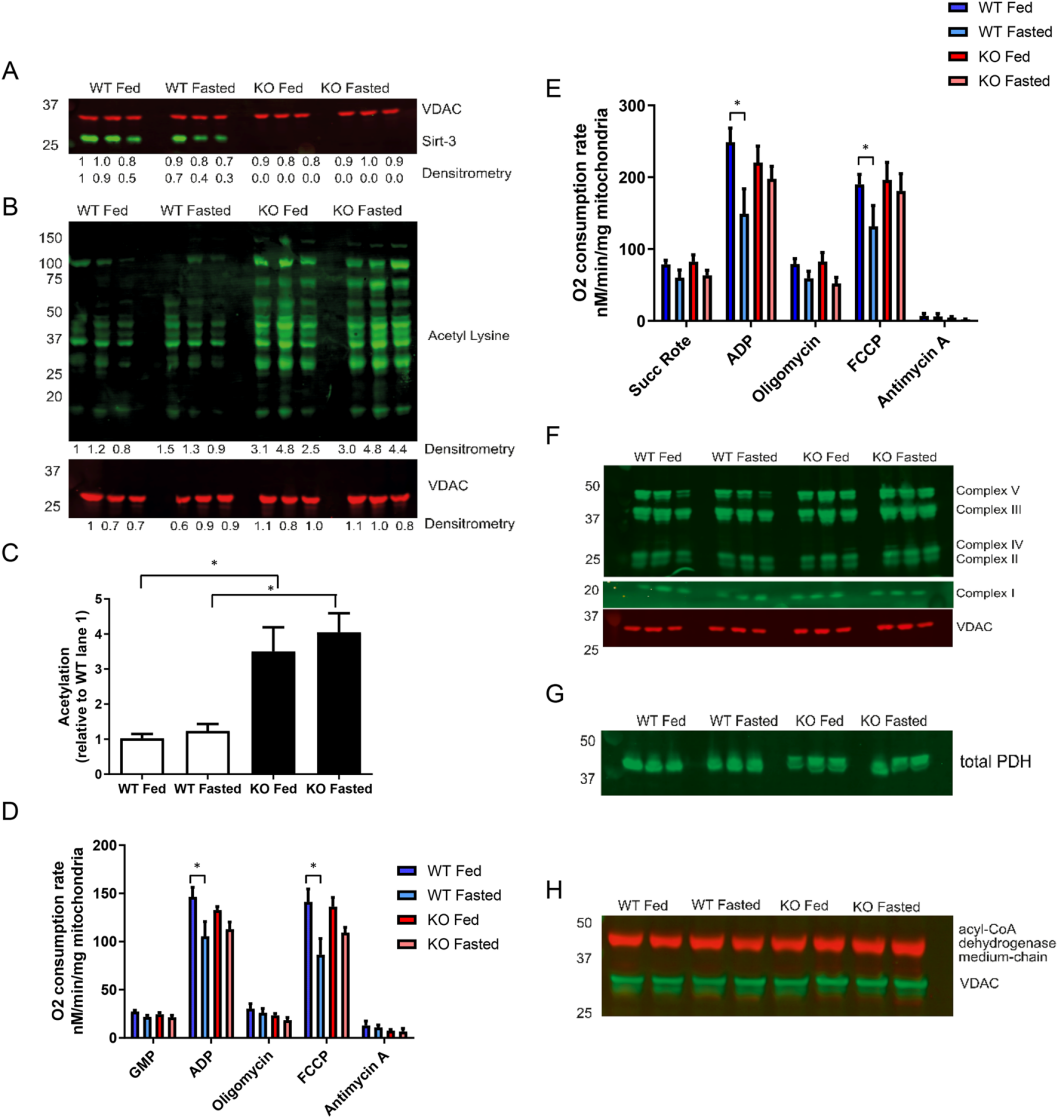
Fasting reduces maximal mitochondrial oxygen consumption in WT, but not SIRT3 KO mitochondria. Cardiac mitochondria from SIRT3 KO mice are hyperacetylated compared to WT mitochondria, with no change induced by transition from fed to fasting. No differences in mitochondrial subunit expression, PDH or Acetyl Co Dehydrogenase are observed. (**A**) No significant changes SIRT3 expression are observed in WT fed vs fasted mice, whilst genetic SIRT3 KO mice lack SIRT3. N = 3/group. Error bars indicate S.E.M. One-Way ANOVA, P > 0.05 (comparing within genotypes). (**B**) SIRT3 KO mitochondrial proteins are significantly hyperacetylated vs WT mitochondrial proteins, whilst the transition from fed to fasting does not alter mitochondrial acetylation. N = 3/group. Error bars indicate S.E.M. One-Way ANOVA with Tukey post-test, *P ≤ 0.05. (**C**) Quantification of pan-mitochondrial acetylation reveals SIRT3 KO mitochondria are significantly hyperacetylated vs mitochondria from WT hearts. N = 3/group. Error bars indicate S.E.M. One-Way ANOVA with Tukey post-test, *P ≤ 0.05. (**D**) Fasting significantly reduces Complex I driven maximal-coupled, and uncoupled respiration in WT cardiac mitochondria, but not SIRT3 KO cardiac mitochondria. N = 6/group. Error bars indicate S.E.M. One-Way ANOVA with Tukey post-test, *P ≤ 0.05. (**E**) Fasting significantly reduces Complex II driven maximal-coupled, and uncoupled respiration in WT cardiac mitochondria, but not SIRT3 KO cardiac mitochondria. N = 6/group. Error bars indicate S.E.M. One-Way ANOVA with Tukey post-test, *P ≤ 0.05. (**F**) The transition from fed to fasting does not alter the expression of mitochondrial subunits isolated from either WT or SIRT3 KO mice hearts. N = 3/group. (**G**) No differences in the expression of pyruvate dehydrogenase (PDH) are observed in cardiac mitochondria isolated from fed/fasted WT and SIRT3 KO mice. N = 3/group. (**H**) No differences in the expression of medium chain acyl-CoA dehydrogenase (MCAD) are observed in cardiac mitochondria isolated from fed/fasted WT and SIRT3 KO mice. N = 3/group.

SIRT3 is known to regulate the mitochondrial acetylome, and the hyperacetylation of mitochondrial proteins in the SIRT3 KO heart has been previously linked to an increased fatty acid respiration, which may explain the differences in metabolic efficiencies observed. Fasting did not alter the expression level of SIRT3 (Fig. 4A) in WT cardiac mitochondria, or the amount of mitochondrial acetylation (when comparing within genotypes). As expected, mitochondrial proteins from SIRT3 KO were significantly hyperacetylated compared to WT mitochondria in both fed and fasted states (Fig. 4B,C). When measuring respiration in isolated mitochondria, the transition from fed to fasted state significantly decreased ADP- driven, and FCCP stimulated maximal respiration capacity in WT mitochondria, with mitochondria isolated from SIRT3 KO hearts unaffected (Fig. 4D,E) for both Complex I (Glutamate, Malate and Pyruvate) and Complex II (succinate and rotenone) driven respiration. No significant changes in the expression of the mitochondrial subunits (Fig. 4F), PDH (pyruvate dehydrogenase, a regulator of glycolysis, Fig. 4G), or acyl-CoA dehydrogenase (a regulator of fatty acid respiration Fig. 4H), were observed when comparing either genotype or fed vs fasted states. This reduction in respiratory capacity may explain the differences in cardiac efficiencies described earlier.

## Discussion

Fasting worsened cardiac outcomes in WT hearts, whilst SIRT3 KO hearts were protected due to a greater cardiac efficiency and adaptation to starvation. At the mitochondrial level, SIRT3 KO mitochondria maintained a greater respiratory capacity when starved, which could be associated with a higher level of acetylation.

In the setting of acute myocardial IRI, inefficient fatty acid oxidation is detrimental due to the higher oxygen burden/ATP molecule produced^33,34^, thus hastening the depletion of oxygen, and exacerbating the ischaemic insult. Upon reperfusion, inefficient utilisation of fatty acids further slows recovery to ionic homeostasis^35^, thus triggering mitochondrial permeability transition pore opening, and cell death. The ability of the SIRT3 KO heart to adapt and utilise ATP more efficiently improved outcomes by maintaining ionic homeostasis for longer, thus reducing the ischaemic burden.

The results presented here contrast with previous studies comparing WT and SIRT3 KO or SIRT3 KD outcomes from cardiac IRI^17,36^. Here, we utilise an in vivo model of IRI, whilst previous studies showed data from glucose-perfused hearts. As demonstrated in our study in Fig. 1, the choice of perfusate is critical to outcomes, which could explain the slight differences in outcomes.

The observed improved fatty acid metabolic efficiency in our study is in agreement with some^9^, but not all^37^ reports where SIRT3 KO increases cardiac fatty acid respiration through lysine hyperacetylation. Acetylation either occurs naturally as a chemical reaction with acetyl CoA^38^, or via protein mediated transfer by GCN5L1. To aid post-translational regulation, SIRT3 deacetylates around 500 mitochondrial proteins^7,39^, including key proteins regulating glucose and fatty acids oxidation (such as PDH, LCAD and β-HAD). Unfortunately, specific protein acetylation was not assessed here, but there is literature reporting cardiac acetylation inhibits PDH^40^, but activates long-chain acyl-CoA dehydrogenase (LCAD), and β-hydroxyacyl-CoA dehydrogenase (β-HAD)^41^ (thus inhibiting glucose oxidation whilst promoting fatty acid oxidation). However, contrary studies performed in liver report that acetylation of LCAD and β-HAD inhibits their activity, reducing fatty acid oxidation^7,42^. The contradictory results could be due to studying differing tissues (heart vs liver), acetylation on differing lysine residues^43^ or differing disease models. Further demonstrating a link between fatty acid respiration and cardiac mitochondrial hyper-acetylation, mitochondrial acetylation increases when WT mice are fed a high-fat diet^8,9^, with a similar increase in mitochondrial acetylation observed when new-borns switch cardiac fuel from glucose to fatty acids^10^. These changes may aid adaption to the predominant fuel source or be a consequence of the increased acetyl CoA flux.

In WT animals, starvation significantly reduced mitochondrial respiratory capacity. No reduction was observed in SIRT3 KO cardiac mitochondria, which may contribute to differences in respiratory efficiency observed between the two starved genotypes in the working heart models. Unfortunately the acetylation status of the complexes was not assessed here, but there are reports suggesting that acetylation can also regulate the activity of the mitochondrial respiratory chain (for review see^44^). The lack of global acetylation differences observed in WT mice between the fed and starved mice is interesting and could be explored further by studying specific site acetylation and SIRT3 activity.

The results presented in our study also challenge the dogma that starvation is protective against acute myocardial IRI. Previous studies demonstrating starvation-mediated cardioprotection were based on data taken from ex vivo hearts perfused with glucose^45^ (similar protection was observed here when fasted WT hearts were perfused with glucose). The absence of fatty acids in the perfusate means the ex vivo conditions failed to accurately replicate the in vivo conditions (i.e. an increased fatty acid respiration). This observation may have clinical implications with most ischaemic episodes occurring in fasted patients i.e. AMIs occurring in the morning are associated with a worse outcome^6^, and patients must undergo a period of starvation prior to elective surgery. Therefore, the data showing that the administration of glucose reduced the susceptibility to acute myocardial IRI with fasting, might suggest that patients could benefit from being infused with, or drinking, a high glucose solution, or treated with fatty acid oxidation inhibitors^46,47^ prior to surgery. Whilst this practise requires clinical validation, it could potentially change current practice and improve patient outcomes.

## Conclusion

Fasting increases infarct size in the hearts of WT mice, which is linked to an increased fatty acid respiration. Hearts from SIRT3 KO animals were resistant to this change as they are better adapted to fatty acid respiration, and thus are metabolically better prepared for the challenge of acute myocardial IRI when fasted.

## Supplementary Information

Supplementary Table 1.

Supplementary Figure 1.

## Data Availability

The datasets used and/or analysed during the current study available from the corresponding author on reasonable request.
