# Supplementary material for: Fasting increases susceptibility to acute myocardial ischaemia/reperfusion injury through a sirtuin-3 mediated increase in fatty acid oxidation
Source: Sci Rep. 2022 Nov 29;12:20551. doi: 10.1038/s41598-022-23847-w (PMC9708654; doi:10.1038/s41598-022-23847-w)

Supplementary Information

**Supplementary Figure 1– Fasting does not alter cardiac infarct size in SIRT3 KO mice.**

a) Fasting does not alter infarct size in *ex vivo* hearts taken from either fed or fasted SIRT3 KO mice, and perfused with 1.2 mM Palmitate and 5 mM glucose. IS as a percentage of the area-at-risk (AAR). N=6/group. Error bars indicate S.E.M.

b) Representative images of short-axis heart slices following staining with tetrazolium to stain the areas of infarction.

c) Fasting does not alter infarct size in *ex vivo* hearts taken from either fed or fasted SIRT3 KO mice, and perfused with 11 mM glucose. IS as a percentage of the area-at-risk (AAR). N=6/group. Error bars indicate S.E.M.

d) Representative images of short-axis heart slices following staining with tetrazolium to stain the areas of infarction.

**Supplementary Table 1– Cardiac metabolomic profile assessed by ^1^H NMR spectroscopy from hearts snap frozen *in vivo* from fed/fasted WT and SIRT3** **KO mice.**

Lactate was the only significantly different metabolite, which was elevated in the hearts of fed and fasted WT mice (vs fed/fasted SIRT3 KO hearts). Analysed by Two Way ANOVA, n=4 for WT fed, n=4 for WT fasted, n=3 for SIRT3 KO fed, n=4 for SIRT3 KO fasted).


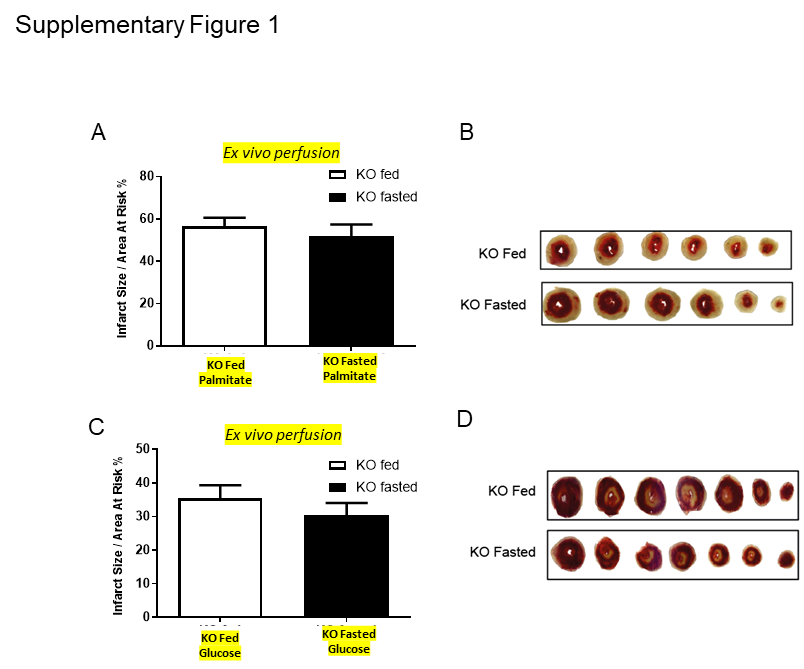


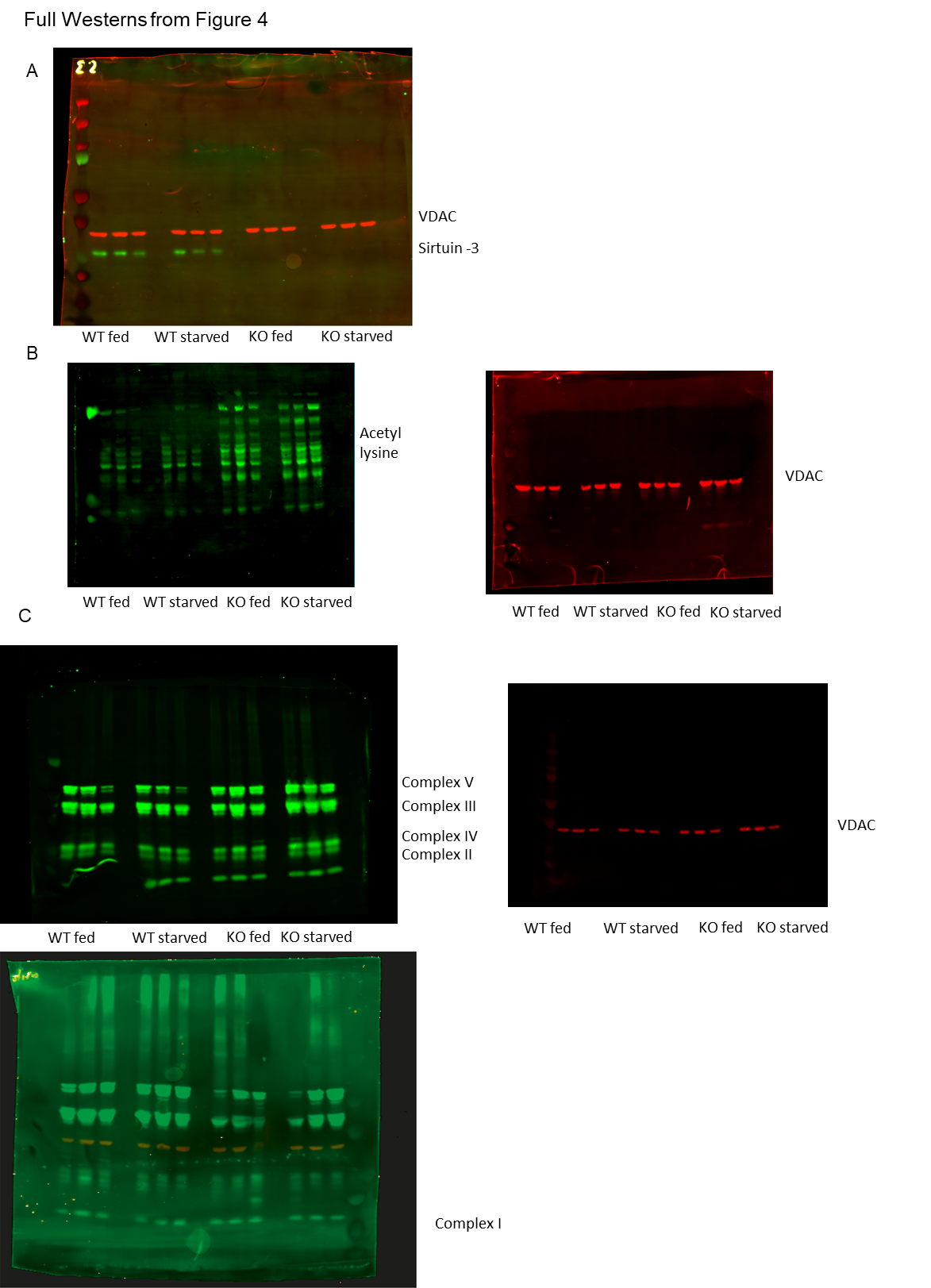


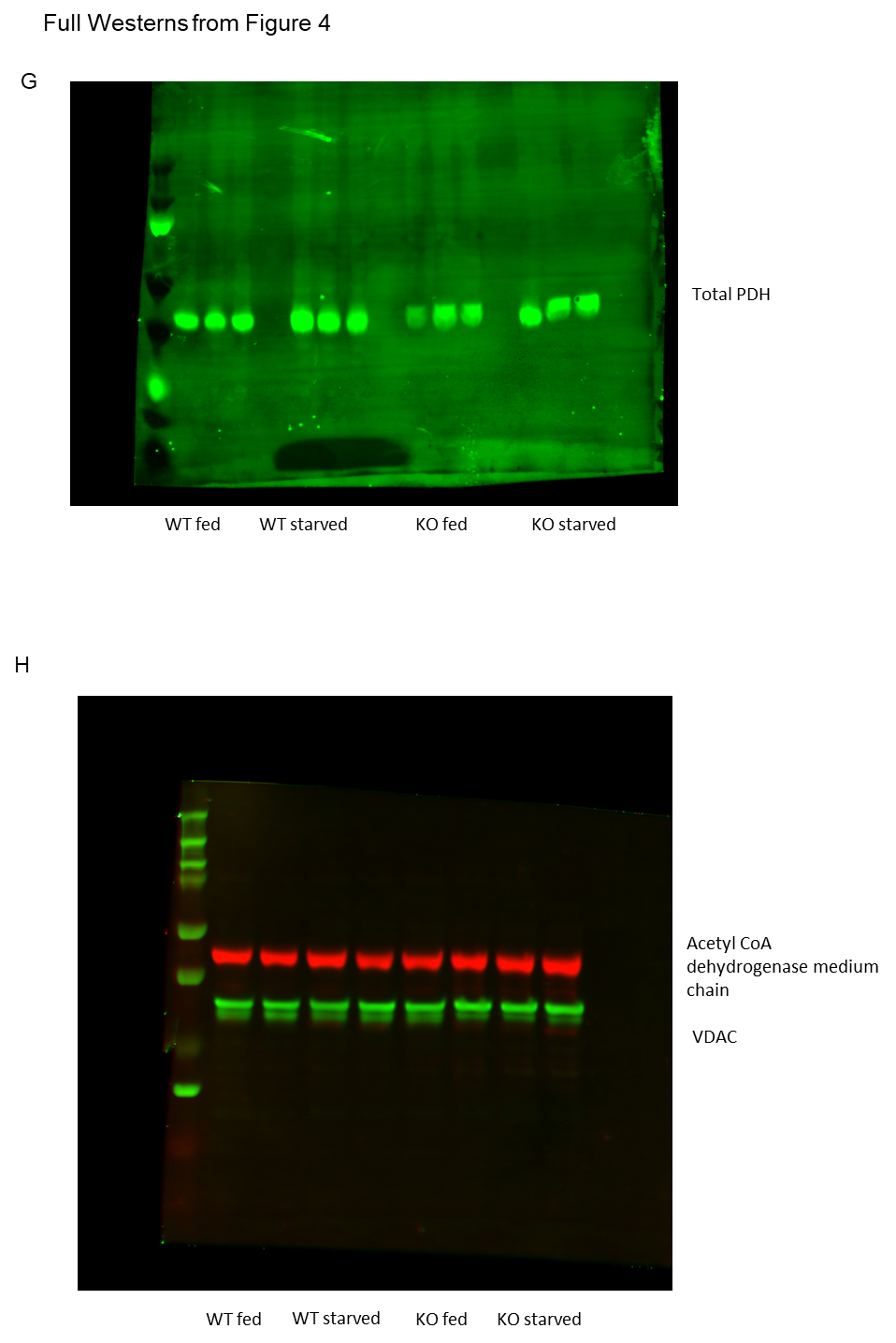

Supplement: Supplementary file 1 — Supplementary Table 1. [file 41598_2022_23847_MOESM1_ESM.docx]
